# An assessment of the continuing medical education needs of US physicians in the management of patients with beta thalassemia

**DOI:** 10.1007/s00277-020-04246-5

**Published:** 2020-09-01

**Authors:** Sylvie Stacy, Sujit Sheth, Brandon Coleman, Wendy Cerenzia

**Affiliations:** 1 CE Outcomes, LLC, Birmingham, AL 35205 USA; 2 Division of Pediatric Hematology and Oncology, Weill Cornell Medical College, New York, NY 10065 USA

**Keywords:** Thalassemia, Quality of life, Transition of care, Continuing educational needs

## Abstract

Patients with beta thalassemia are benefitting from longer life expectancies, highlighting the importance of appropriate transition from pediatric to adult care. Data are limited regarding continuity of care and adult hematologists’ management of patients with beta thalassemia. We conducted a survey of practicing US hematologists to identify practice gaps, attitudes, and barriers to optimal patient management among US-practicing hematologists. A total of 42 responses were collected, with 19 (45%) practicing at a beta thalassemia center of excellence (CoE). Nearly 90% of CoE physicians said they had a transition protocol or plan in place versus 30% of non-CoE physicians. Most physicians said parents should remain actively involved in medical visits. Adherence was rated as the most important patient education topic during transition. The most significant barrier cited was patient reluctance to transition away from pediatric care. Physicians in CoEs as compared with non-CoE physicians reported greater knowledge of beta thalassemia and familiarity with butyrates, gene therapy, and luspatercept. Highly rated topics for beta thalassemia-focused CME activities included management of complications and clinical trial updates. These findings suggest practice gaps and barriers to optimal care in the transition from pediatric to adult care, the ongoing management of adult patients, knowledge of the disease state, and familiarity with emerging treatments. Differences CoE vs non-CoE physician responses suggest variations in knowledge, practice, and attitudes that may be helpful in tailoring CME activities to different learner audiences. The small sample size used in some sub-analyses may not be representative of all hematologists treating beta thalassemia patients.

## Introduction

Beta thalassemia is an inherited red cell disorder with a broad clinical spectrum, the hallmark being ineffective erythropoiesis. It represents a growing global health concern, affecting approximately 23,000 births each year [1]. In the USA, more than 1000 individuals are living with beta thalassemia major, the most severe form of the disease [2]. Conventional treatment includes regular blood transfusions, along with chelation therapy to manage the burden of iron overload that occurs secondary to transfusions [3].

Medical care for beta thalassemia traditionally has been focused on younger patients, with the majority of thalassemia treatment centers being pediatric centers/hospitals [4]. However, as improvements in treatment continue, patients are benefitting from longer life expectancies with fewer complications, highlighting the importance of appropriate transition from pediatric to adult care to provide continuity and ensure the best possible patient outcomes [5, 6].

There are limited data regarding the practice of pediatric hematologists transitioning patients from pediatric to adult care and adult hematologists’ management of patients with beta thalassemia. A review of medical literature indicates that transitioning is often marked by an abrupt transfer, leaving youths with hemoglobinopathies unprepared to move to adult care [7]. Moreover, the transition happens in adolescence, an age at which adherence to medical therapy (i.e., iron chelation) begins to decline [8], and before the adolescent is emotionally prepared to handle it, which may in part explain poor adherence [6].

Accordingly, research is needed to better understand practice patterns with regard to care transition and adult care of patients with beta thalassemia. Surveys, ideally with the inclusion of case vignettes, are one effective way to obtain rapid feedback from a broad group of clinicians. Case vignettes have gained considerable support for their value in predicting healthcare provider practice patterns, based in part on data demonstrating that they (as compared with chart review and standardized patients) are a valid and comprehensive method to measure processes of care in actual clinical practice [9–11]. Case vignettes are more cost-effective and may be viewed by respondents as less intrusive compared with other means of measurement.

To better elucidate issues in care transition and adult care of patients with beta thalassemia, we conducted a survey of practicing hematologists in the USA. This survey was designed to obtain data on current practice and identify potential practice gaps, attitudes, and barriers to optimal patient management that could be targeted through continuing medical education (CME) activities.

## Methods

A survey instrument was developed to investigate hematologist approaches diagnosis and management of patients with beta thalassemia, as well as their transition from pediatric to adult care. The survey, which was designed to be completed in 15–20 min, included 2 patient case vignettes with associated questions to assess management choices and questions to assess knowledge, attitudes, and perceived barriers to optimal patient care. A small monetary incentive ($75 online gift card) was offered for participation. Email invitations to participate in the surveys were sent to US hematologists, with inclusion criteria ensuring that each respondent was currently practicing and actively managing at least one adult patient with beta thalassemia. For analysis, quotas were placed to ensure approximately 45% of the responses came from hematologists who are currently practicing in beta thalassemia center of excellence (CoE), defined as one of the six centers in the USA designated for clinical excellence in thalassemia by the National Cooley’s Anemia Foundation and previously funded by the CDC [4].

Physician practices and attitudes toward the pediatric to adult transition were assessed using patient case scenarios (Table 1). These included an 18-year-old male with transfusion-dependent beta thalassemia who is about to start college. Specific questions were asked about initial patient transition, parent involvement, important topics for education of transitioning patients, and barriers to transition. Physician practices and attitudes regarding the management of adult patients were assessed using a second patient case scenario regarding otherwise healthy 30-year-old woman with non-transfusion-dependent beta thalassemia. Specific questions were asked about testing, treatment goals, the initial visit, hemoglobin levels and transfusion, familiarity with emerging treatments, and barriers to optimal management.

**Table 1:** Patient cases included in a physician survey

Scenario	Description
1. Transitioning from pediatric to adult care for transfusion-dependent beta thalassemia	An 18-year-old male with transfusion-dependent beta thalassemia presents to your office as a new patient, accompanied by his mother. He is referred by his pediatric hematologist, who has been managing the boy’s beta thalassemia since infancy. He is a high school senior and is planning to start college at a local university in the fall. He has no complaints. He is on iron chelation therapy and has a regular follow-up with subspecialists in pediatrics including endocrinology and cardiology.
2. Management of adult patient with non-transfusion-dependent beta thalassemia	A 30-year-old woman with non-transfusion-dependent beta thalassemia presents for routine follow-up. She has no new symptoms but continues to complain of fatigue and malaise. She is otherwise healthy and has never required a transfusion. She takes folic acid, is not on medications, and has not had comprehensive testing in 3 years. Initial lab workup reveals a hemoglobin of 10.6 g/dL.

### Statistical analysis

Data were collected using an online survey platform (Qualtrics). Survey data were compiled and analyzed with IBM SPSS Statistics 25. Descriptive statistics, such as frequencies and means, were calculated on all items to examine overall responses and related trends among the survey items. Inferential statistics, including *t* tests and chi-squares, were conducted to analyze and interpret differences between hematologists practicing in a beta thalassemia CoE. Differences between groups were considered statistically significant at *P* ≤ .05.

### Demographics

A total sample of 42 hematologists was collected with 19 (45%) practicing at a center designated for clinical excellence in thalassemia (center of excellence; CoE). Respondent demographics (Table 2) indicate that the majority of CoE physicians (84%) reported their practice type as academic based, while conversely, the majority of non-CoE physicians (74%) reported being in community-based practice. The mean number of years in practice was 23 for CoE physicians and 29 for non-CoE physicians.

**Table 2:** Respondent demographics

	COE physicians (*n* = 19)	Non-COE physicians (*n* = 23)
Practice type, %		
Community based	16	74
Academic based	84	26
Practice location, %		
Urban	58	57
Suburban	42	39
Rural	0	4
Number of adult patients* personally managed for beta thalassemia, %		
1–5 adult patients	37	44
6–15 adult patients	16	21
16–25 adult patients	16	17
25+	30	17
Time in practice, years (mean)	23	29

CoE, centers of excellence

*18 years of age and older

## Results

### Knowledge of beta thalassemia

Physicians not affiliated with CoEs generally rated their knowledge of beta thalassemia lower than CoE physicians on pathophysiology and genetics (mean rating on 1–5 Likert scale, 3.8 vs 4.3 for non-COE and COE physicians, respectively), clinical characteristics (3.8 vs 4.5), anemia management (3.8 vs 4.4), and clinical trials (4.2 vs 4.6). While physicians generally report that they were knowledgeable regarding clinical characteristics and pathophysiology of the disease, few appropriately recognized the main mechanism of ineffective erythropoiesis in beta thalassemia. When asked to identify the main mechanism of ineffective erythropoiesis, many CoE and non-CoE physicians incorrectly selected “formation of unstable beta globin homotetramers,” characteristic of alpha thalassemia (47% and 39%, respectively), while about one-third correctly selected “precipitation of excess free alpha chains in red blood cell precursors” characteristic of beta thalassemia (37% and 39%). Physicians who correctly identified the mechanism of action of beta thalassemia indicated higher mean knowledge of pathophysiology and genetic basis of disease (4.38 vs 3.85) and current best practices in anemia management (4.38 vs 3.88). Both CoE and non-CoE physicians identified abnormal liver function and osteoporosis as prevalent clinical issues in patients with beta thalassemia

### Transitioning from pediatric to adult beta thalassemia care

#### Transition plans

**Fig. 1:**
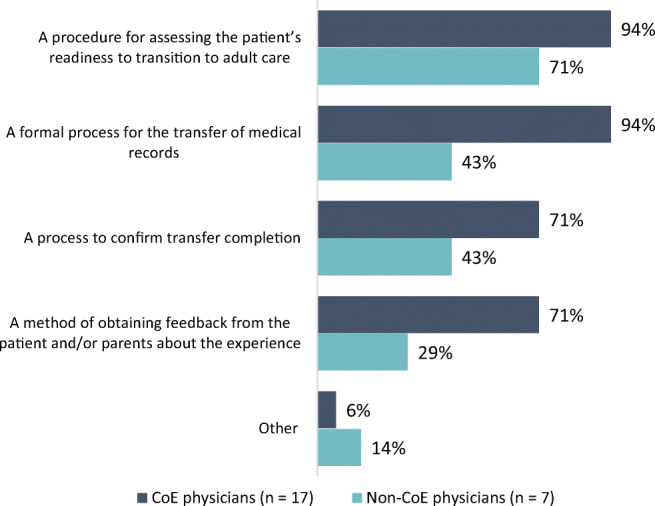
Components included in the transition protocol or plan

**Fig. 2:**
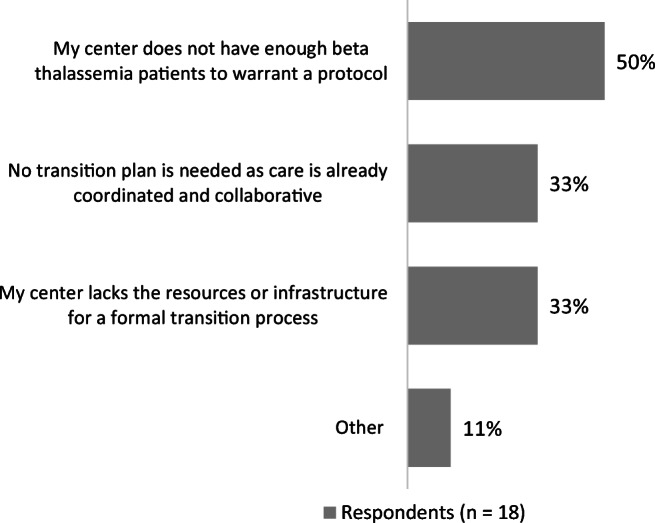
Reasons for no transition protocol

While 89% of CoE physicians said they had a protocol or plan in place to transition pediatric patients to adult care, only 30% of non-CoE physicians had such a protocol. Among physicians with a protocol in place, most (94% and 71% of CoE and non-CoE physicians, respectively) said that it included a procedure to assess the patient’s readiness to transition to adult care (Fig. 1). Other key components of the protocol were endorsed more often by CoE physicians than non-CoE physicians, including a formal process to transfer medical records (94% vs 43%), a process to confirm transfer completion (71% vs 43%), and a method to obtain feedback from the patients or parents about the transition experience (71% vs 29%). Reasons cited for a lack of transition protocol included lack of resources or infrastructure, too few patients, only seeing adult patients, and a perception that the care is already so coordinated and collaborative that no specific protocol is needed (Fig. 2).

#### Patient case assessment

##### Initial patient transition

Physicians described the most relevant factors, ranked in order of importance, as adherence to the current medication regimen, knowledge of the disease, personal goals, developmental maturity, and emotional status. When asked what topics they would provide to the patient or parent at the initial transition visit, nearly all said they would provide information on patient expectations or responsibilities (100% of CoE and 91% of non-CoE physicians) and information about the center and patient services (95% of CoE and 83% of non-CoE physicians), while fewer said they would discuss insurance issues at that first visit (47% of CoE and 57% of non-CoE physicians).

##### Parent involvement

The majority of CoE and non-CoE physicians (53% and 65%) said they would recommend that parents remain actively involved in medical visits, while fewer (42% and 22%) said they would recommend parents “fade” their involvement in medical visits as the patient transitions. A substantial minority of physicians (5% and 13%) selected “other” and discussed the need for individual flexibility depending on the patient (e.g., “Every person is different. Listening is the key to a good physician-patient relationship, establishing trust, and getting the best outcomes.”) When asked for specifics, those recommending active involvement of parents offered reasons including increased likelihood of adherence to treatment, financial dependence, and emotional dependence, while those recommending a fade cited the importance of autonomy, patient well-being, and reduced caregiver burden, among others.

##### Patient education

Adherence was rated as the most important patient education topic during the transition from pediatric to adult care (79% and 74% of CoE and non-CoE physicians). Other important topics included disease complications (53% and 65%) and patient expectations for treatment (58% and 30%). Few physicians felt that clinical trial enrollment, results of clinical trials, or disease pathophysiology were important patient education topics.

##### Barriers to transition

According to both CoE and non-CoE physicians, the most significant barrier in the transition to adult care is patient reluctance to transition away from pediatric care, while the second-most significant barrier is finding a provider. The third most significant barrier for CoE physicians is lack of support resources, while for non-CoE physicians, it is insurance/cost.

#### Management of adult patients with beta thalassemia

##### Baseline testing

For a patient with non-transfusion-dependent beta thalassemia who had not recently had comprehensive testing, the majority of CoE and non-CoE physicians indicated they would order endocrine studies such as thyroid function, glucose tolerance, and calcium level (95% and 87%), liver function testing (89% and 96%), and kidney function testing (84% and 78%) bone health studies such as a dual-energy X-ray absorptiometry scan (84% and 74%), cardiac studies (79% and 65%), and iron burden assessment (79% and 65%). Somewhat fewer physicians (roughly half) said that genetic counseling and viral testing would be warranted.

##### Treatment goals

Maximizing quality of life was the top-ranked treatment goal for both CoE and non-CoE physicians. The next most highly ranked goal for CoE physicians was minimizing anemia-related symptoms, followed by monitoring for complications at number three and correcting anemia at number four; conversely, correcting anemia was the second-highest ranked goal for non-CoE physicians, followed by complication monitoring and minimizing anemia symptoms.

##### Initial visit

**Fig. 3:**
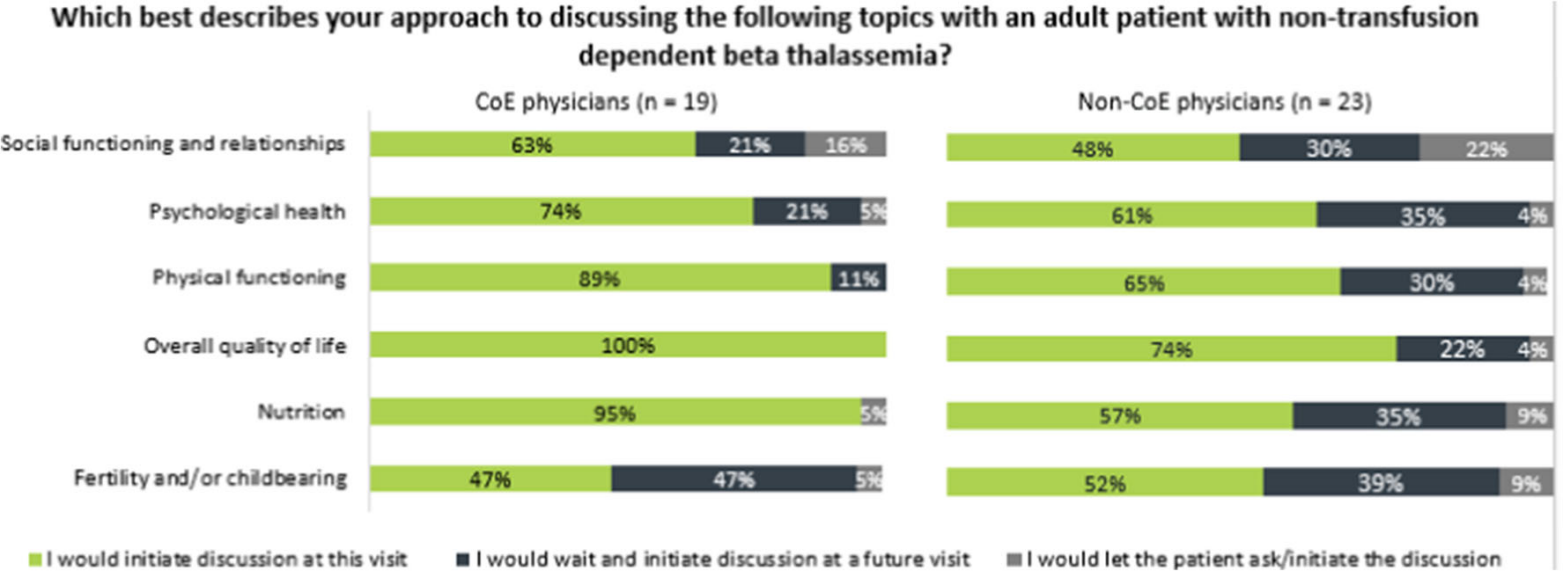
Topics to address at initial visit, adult patient with non-transfusion-dependent beta thalassemia

Almost all physicians at CoEs said that at the initial visit, they would initiate discussion of overall QoL (100%), nutrition (95%), and physical functioning (89%), whereas a larger proportion of non-CoE physicians said they would initiate these discussions at a future visit; as a result, the percentage of non-CoE physicians saying they would initiate discussion of QoL, nutrition, and physical functioning were, respectively, 74%, 57%, and 65% (Fig. 3).

##### Hemoglobin levels and transfusion

Most respondents said that in adults with beta thalassemia, they would begin transfusions at 7–8 g/dL (63% and 52% of CoE and non-CoE physicians, respectively). Once transfusions are started, most physicians said they would target a hemoglobin of 9–10 g/dL (42% and 26%) or 10–11 g/dL (21% and 30%). Iron overload was the most frequently cited challenge related to blood transfusion, followed by adherence or compliance with therapy, time and convenience issues (e.g., related to adjusting work and school schedules), and red blood cell alloimmunization. Other challenges included infection risk, liver damage, increasing cost, transfusion reactions, and quality of life.

##### Familiarity with emerging treatments for beta thalassemia

**Fig. 4:**
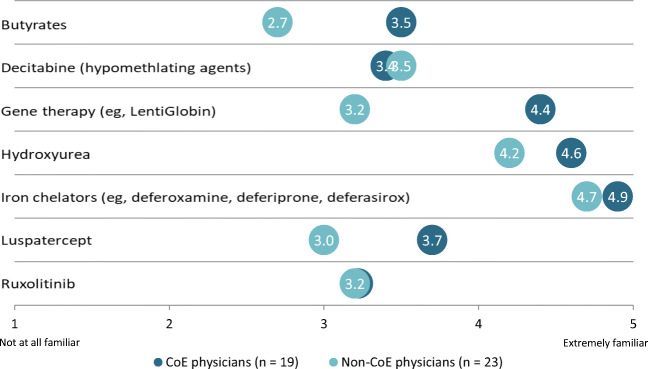
Self-reported familiarity with current and emerging therapies for beta thalassemia

Physicians generally self-rated their knowledge of ongoing clinical trials and investigational approaches as high, with a mean score of 4.2 and 4.6 for CoE and non-CoE physicians, respectively, on a scale of 1 to 5 (from “not at all knowledgeable” to “extremely knowledgeable”). Physicians in CoEs as compared with non-CoE physicians reported higher familiarity with butyrates (mean scores of 3.5 and 2.7, respectively), gene therapy (mean scores of 4.4 and 3.2), and luspatercept (mean scores of 3.7 and 3.0). Familiarity with ruxolitinib was moderate with a mean score of 3.2 for both CoE and non-CoE physicians. Familiarity was generally high for hydroxyurea (mean scores of 4.6 and 4.2), iron chelators (mean scores of 4.9 and 4.7), and low for hypomethylating agents; e.g., decitabine (mean scores of 3.4 and 3.5) (Fig. 4).

##### Barriers to optimal management in adult patients

Based on mean scores on a 5-point Likert scale, with 1 being not at all challenging to 5 extremely challenging, the barriers most frequently attested to as significant included a lack of disease-modifying therapies with 3.8 and 4.0 for CoE and non-CoE physicians, respectively, the need for frequent infusions (3.6 and 4.1), and the need for frequent visits to multiple healthcare providers (3.8 for both CoE and non-CoE physicians). Both groups of physicians found co-management of patients with beta thalassemia moderately challenging, with mean scores of 2.8 and 2.9, respectively. When asked about specific barriers to effective co-management, CoE physicians found logistical difficulties in coordinating care to be more significant of a barrier than non-CoE physicians, with mean scores of 3.9 and 3.3, respectively, on a scale of 1 to 5 (not at all significant to extremely significant).

#### Educational resources and learning preferences

##### Educational resources for patients transitioning to adult care

More than half of physicians (53% of CoE and 61% of non-CoE) said they recommend specific educational resources for patients transitioning to adult care. In aggregate, roughly 80% of CoE and non-CoE physicians said they recommend patient support groups, and 80% said they would provide a referral to a social worker. More than 60% said they would provide written handouts or information about transitioning to adult care, while 50% said they would provide internet-based resources.

##### Educational resources for adult patients

Seventy-nine percent of CoE physicians and 64% of non-CoE physicians said they recommend support groups to adult patients with beta thalassemia. The most frequently recommended support groups for CoE and non-CoE physicians, respectively, were Cooley’s Anemia Foundation (73% and 71%), Thalassemia Support Foundation (60% and 57%), local or regional support groups (47% and 43%), and Thalassemia International Federation (40% and 36%). In addition, most physicians (77% to 84%) said they recommend printed educational materials and relevant websites or online resources for adult patients with beta thalassemia.

##### CME engagement and topics for future education

Of CoE and non-CoE physicians, respectively, 53% and 22% said they had participated in thalassemia-focused CME programs in the past 12 months. Reasons given for not participating in CME activities generally focused on issues such as lack of opportunity (e.g., “I haven’t found one in my region”) or limited time to participate. Physician willingness to participate in beta thalassemia-focused CME activities was relatively high, with mean scores of 3.7 for CoE physicians and 3.9 for non-CoE physicians on a scale ranging from 1 (not at all likely) to 5 (extremely likely). The two topics respondents would most like to see in future educational opportunities are prevention and management of complications (84% and 70% of CoE and non-CoE physicians, respectively) and updates on clinical trials and investigational approaches (68% and 78%). Transitioning patients from pediatric to adult care was selected as a top of interest by 53% of CoE physicians versus 22% of non-CoE physicians.

## Discussion

Results of this survey provide insights into current US physician practices and attitudes regarding beta thalassemia. The findings suggest practice gaps and barriers to optimal care in the transition from pediatric to adult care, the ongoing management of adult patients, knowledge of the disease state, and familiarity with emerging treatments with the potential to improve clinical outcomes. In addition, we provide data describing the utilization of educational resources for patients and providers, and the learning preferences for physicians who provide care for patients with this condition. By obtaining responses from CoE and non-CoE physicians and analyzing them separately, we were able to identify apparent differences in knowledge, practice, and attitudes that may be helpful in tailoring continuing education activities to different learner audiences; however, we acknowledge that the small sample size used in some sub-analyses may not be representative of all hematologists treating beta thalassemia patients.

Our key findings with regard to knowledge of beta thalassemia demonstrate a significant deficiency in knowledge with regard to clinical characteristics and disease pathophysiology in over half the providers surveyed. Both CoE and non-CoE physicians generally self-reported a relatively high level of knowledge regarding clinical characteristics and pathophysiology of the disease, but only about one-third appropriately recognized the pathophysiology of beta thalassemia, suggesting a need for further education to support foundational knowledge of this disease. This knowledge forms the basis for a rational approach to treatment, including transfusion therapy and iron chelation.

With regard to care transitions, that majority of physicians in CoEs report having a protocol for transitioning patients to adult care, compared with less than one-third of non-CoE physicians. The difference between CoE and non-CoE hematologists in their reasons for not having a transition protocol suggests a need to improve awareness of the benefits protocols for non-CoE clinicians and to offer practical strategies for implementation outside of CoEs.

Although there is little data on care transition in beta thalassemia, the literature on sickle cell disease (SCD) indicates that the transition from pediatric to adult health services is frequently challenging for adolescents and young adults, which may be due in part to barriers that include negative experiences in the emergency department, adolescent skills, and sociodemographic factors; by contrast, factors that may increase the success of transition include a positive relationship with the provider, developmental maturity, and family support [12]. In a retrospective evaluation of factors associated with the success of a pediatric SCD transition process, several factors were found to increase the risk of unsuccessful transition, including travel distance to the adult SCD center, older age at first transition, and milder disease severity increased risk of unsuccessful transition [13]. Specific interventions to improve the success of transition have also been evaluated. In a systematic review including 5 studies conducted in specialty clinics, 3 studies found that the intervention was associated with an increased transfer rate [14]. A review study provided some evidence that interventions designed to improve the transition of care for adolescents from pediatric to adult health services have the potential to improve certain outcomes, including patients’ knowledge of their condition, and improvements in self-efficacy and confidence [15].

The substance of the transition deserves further education with regard to the conduct of the initial transition visit and the ongoing involvement of parents. While the first visit represents an opportunity to assess readiness for transition through consideration of developmental maturity, emotional status, and other factors, physicians in this survey ranked adherence to treatment as the most important factors to assess at this visit. While the “optimal” approach for this visit may be subjective, assessing readiness for transition should ideally be the most important factor, as adherence for the transitioning patient, moving forward, will depend on patient readiness, knowledge of the disease, personal goals, maturity, and emotional status.

Physicians had mixed perspectives on whether parents should remain active or fade their involvement in the transition of their child to adult care. Some physicians noted that it would be useful to have a protocol with room for personalization, based on each patient and their wishes. When asked, some patients may want their parents to remain closely engaged, while others (e.g., children leaving for college) may desire greater autonomy. Some strategies to ease and personalize the transition may include an independence plan, coping strategies, and involvement of social workers.

Patient reluctance was identified in this survey as the most significant barrier physicians faced in transitioning a patient from pediatric to adult care. This finding is consistent with literature documenting the uneasiness among adolescents with chronic illnesses who are faced with emotions such as anxiety or fear when entering a new environment and leaving the care of trusted pediatric providers [16, 17]. Given the potentially traumatic nature of transition, education should include consideration of programs geared toward improving the transition process.

Physician responses regarding the management of adults with beta thalassemia in this survey suggest that most are performing basic laboratory testing at appropriate times and that maximizing quality of life is a top priority; nevertheless, only 42% of CoE physicians and just 22% of non-CoE physicians reporting the use of a standardized tool such as the 36-Item Short Form Survey (SF-36). Evaluating each patient with a standardized tool is ideal and allows clinicians to see and evaluate trends in quality of life in a longitudinal manner [18]. Beyond the overall quality of life, conversations about social functioning, psychological health, physical functioning, and nutrition are all important and should be individually tailored for patients at the appropriate time and should be based on the patient’s goals and expectations of treatment; unfortunately, expertise in some or all of these topics may be lacking at community-based centers.

The key to optimizing treatment and thereby maximizing quality of life is an appropriate transfusion and chelation program, including individually tailoring iron overload management based on patient assessment. The survey results provide some insights into current treatment practices and familiarity with emerging therapies. Most CoE and non-CoE physicians said they would begin transfusions at a hemoglobin level of 7–8 g/dL; however, only about 60% of both CoE and non-CoE physicians would target to maintain hemoglobin levels above 9 g/dL, which is recommended in thalassemia-specific guidelines [6] and is specifically different for these patients because of the underlying ineffective erythropoiesis. There is also still some room for a more comprehensive approach to iron overload assessment and cardiac evaluation. While the majority of physicians identified resources such as the Cooley’s Anemia Foundation and the Thalassemia International Federation, which have extensive educational resources and guidelines on their websites, patients were not being managed according to these recommendations. Furthermore, while physicians felt very knowledgeable about ongoing clinical trials and investigational approaches to treatment, self-reported knowledge of investigational therapies was lower.

### Recommendations for future educational focus

Levels of participation in CME activities in this survey were surprisingly low, given the aforementioned identified areas for improvement (disease knowledge, familiarity with treatments, transition, adult care) and continued improvements in the care of patients with beta thalassemia. Only about half of physicians at CoEs and one-quarter of those not at CoEs reported participation in CME/CE on the topic of beta thalassemia in the past 12 months.

Nevertheless, physicians reported a high likelihood to participate in CME, if it were available. Respondents indicated that complication prevention and management and clinical trial updates were topics of interest for future education, though the gaps in knowledge and practice documented in this survey indicate that a more comprehensive scope of education would be warranted. The topics of clinical characteristics and pathophysiology were selected by less than one-quarter of physicians; however, survey results suggest that inclusion of these topics as part of future education would be beneficial.

Taken together, these findings suggest a need for education in all aspects of beta thalassemia, the effectiveness and relevance of which would be enhanced by a focus on increasing knowledge of beta thalassemia, improving the pediatric to adult care transition, enhancing adult care through assessment of the quality of life and patient functioning, and raising awareness of emerging agents. Education should seek to improve awareness and knowledge of beta thalassemia pathophysiology, clinical characteristics, and management. Special attention should be paid to elements of planning transition for those providers who do not have a transition protocol and should describe approaches to tailoring parental involvement based on patient preferences, while addressing key barriers to transition, such as the lack of support resources. Education can also emphasize the benefits of standardized tools for assessing the quality of life, and the importance of initiating discussion with patients regarding quality of life, psychological well-being, and social functioning. Finally, it should include new and emerging treatments, especially for clinicians not in CoEs, who may not be as familiar with these treatments as those who practice in CoEs.

## Conclusions

This survey reveals substantial gaps in knowledge of beta thalassemia and accessing of currently available educational resources among practicing hematologists, including those who practice at CoEs and those who do not. It is likely that self-learning is not as widely used as believed, and physicians may need to have more didactic learning in order to be up-to-date on current concepts and be able to apply them to patient care, representing opportunities for education. As with most chronic lifelong diseases which manifest early in life, transition from pediatric to adult care remains a major challenge. This survey identifies potential ways of helping physicians with this process. Given the self-reported likelihood to participate in future education on beta thalassemia, education that is developed to focus on the educational needs identified in this survey can work to close the gaps in knowledge and alignment of practice with guidelines.
